# Gut Microbiota-Derived Butyric Acid Alleviates Glucocorticoid-Associated Osteonecrosis of the Femoral Head via Modulating Inflammatory Cytokines in Bone Marrow Mesenchymal Stem Cells

**DOI:** 10.1155/mi/8742817

**Published:** 2025-06-05

**Authors:** Shuai He, Hao Chen, Hongzhong Xi, Guangquan Sun, Bin Du, Xin Liu

**Affiliations:** ^1^Department of Orthopedics, The Affiliated Hospital of Nanjing University of Chinese Medicine, Nanjing 210029, China; ^2^Department of Orthopedics, Jiangsu Province Hospital of Chinese Medicine, Nanjing 210029, Jiangsu, China

**Keywords:** BMSCs, bone metabolism, butyrate acid, GA-ONFH, gut microbiota

## Abstract

**Background:** The role of gut microbiota and its metabolites in regulating bone metabolism has been well established, with inflammatory immune responses potentially playing a critical role. Glucocorticoid-associated osteonecrosis of the femoral head (GA-ONFH), caused by high-dose glucocorticoid use for inflammatory or immune-related diseases, is a prevalent condition of bone metabolic imbalance. However, the regulatory role and mechanisms of gut microbiota and its metabolites in the development and progression of GA-ONFH remain unclear. This study aims to investigate the intervention effects of gut microbiota and its metabolite butyric acid on GA-ONFH through a series of multi-omics *in vitro* and *in vivo* experiments.

**Methods:** Sprague Dawley rats were randomly divided into four groups. The gut microbial composition of the groups was analyzed through 16S rDNA sequencing. Targeted metabolomics was employed to assess differences in short-chain fatty acids (SCFAs) among the groups. Butyric acid, identified as a key differential metabolite, was then selected for further exploration of its effects on bone marrow mesenchymal stem cells (BMSCs) and GA-ONFH rat models through *in vitro* and *in vivo* experiments.

**Results:** 16S rDNA sequencing revealed alterations in gut microbiota structure in GA-ONFH rats. Micro-CT and HE staining demonstrated that depletion of gut microbiota with broad-spectrum antibiotics prior to GA-ONFH modeling exacerbated the disease's development. In contrast, fecal microbiota transplantation (FMT) was shown to alleviate GA-ONFH progression. Targeted metabolomics indicated that FMT mitigated the reduction in butyric acid levels induced by dexamethasone (DXM). Subsequent *in vitro* cell experiments confirmed that butyric acid promotes BMSC proliferation, migration, and osteogenic differentiation. RNA sequencing revealed that butyric acid regulates T cell-mediated inflammatory cytokine genes in BMSCs, while Western blot and immunofluorescence assays confirmed that butyric acid modulates the expression of TNF-α and IL-2/IL-4 in BMSCs. Finally, *in vivo* experiments demonstrated that butyric acid supplementation attenuated the progression of GA-ONFH and improved the expression of inflammation-related cytokines in femoral head tissue.

**Conclusions:** Our study demonstrates that gut microbiota depletion exacerbates GA-ONFH, while FMT restores butyric acid levels and alleviates disease severity. Butyric acid reduced the expression of TNF-α and IL-2 while increasing the level of IL-4 *in vivo* and *in vitro*, thereby improving the local inflammatory environment of the femoral head and alleviating the progression of GA-ONFH. These findings highlight that reduction in butyric acid levels due to gut microbiota dysbiosis is a crucial factor in the progression of GA-ONFH.

## 1. Introduction

Glucocorticoid-associatedosteonecrosis of the femoral head (GA-ONFH) is a severe and progressive musculoskeletal condition characterized by impaired vascular supply and subsequent necrosis of the femoral head [1]. Despite advances in orthopedic surgery and pharmacological interventions, the pathogenesis of GA-ONFH remains poorly understood, leading to limited efficacy in prevention and treatment strategies. The condition is particularly prevalent among individuals receiving high-dose or long-term glucocorticoid therapy, highlighting the need for an in-depth understanding of its underlying mechanisms.

Glucocorticoids (GCs) are widely used as potent anti-inflammatory and immunosuppressive agents in the management of various autoimmune and inflammatory diseases [2, 3]. However, their use is associated with significant adverse effects, including osteoporosis, impaired angiogenesis, and dysregulation of immune homeostasis [4, 5]. GCs can suppress the secretion of critical cytokines, modulate T-cell function, and disrupt the balance between proinflammatory and anti-inflammatory pathways, contributing to tissue injury and delayed healing [6, 7]. In the context of GA-ONFH, GCs exacerbate vascular damage and induce apoptosis in osteoblasts and osteocytes, further compromising bone integrity [8, 9]. Despite these insights, the precise role of glucocorticoid-mediated immune modulation in GA-ONFH pathogenesis remains unclear.

Previous studies have predominantly focused on systemic effects and metabolic pathways in GA-ONFH [10, 11], while the local cytokine microenvironment at the site of osteonecrosis has received limited attention. Cytokines play a pivotal role in orchestrating bone metabolism, vascular remodeling, and immune responses, suggesting that their dysregulation may be a critical contributor to the progression of GA-ONFH [12]. Emerging evidence highlights the gut microbiota as a regulator of systemic and local immune homeostasis, producing metabolites such as short-chain fatty acids that influence cytokine expression and immune cell differentiation [13, 14]. However, the interplay between gut microbiota-derived metabolites and the cytokine milieu in GA-ONFH has yet to be fully elucidated.

This study aims to investigate the effects of gut microbiota and its metabolic products on the cytokine express in GA-ONFH through multi-omics analyses combined with *in vivo* and *in vitro* experiments. By focusing on cytokine regulation, we seek to identify novel mechanistic insights and potential therapeutic targets for the prevention and management of GA-ONFH. This research provides a foundation for leveraging gut microbiota modulation as a promising strategy to mitigate the adverse effects of GCs on bone health.

## 2. Materials and Methods

### 2.1. Animals

All experimental procedures involving animals were conducted in accordance with guidelines approved by the Animal Ethics Committee of Nanjing University of Chinese Medicine (Approval No. 202306A059). Male Sprague Dawley (SD) rats (aged 6 weeks; body weight 200–250 g) were purchased from Hangzhou Medical College Co., Ltd. (Hangzhou, China). Animals were housed under specific pathogen-free environment with controlled temperature (22 ± 1°C), relative humidity (50 ± 5%), and a 12-h light/dark cycle, with ad libitum access to food and water. All efforts were made to minimize the suffering of the animals involved in the experiments, such as providing a clean and comfortable environment with adequate food, drinking water, and space; performing animal experiments with standardized practices; adequately anesthetizing with pentobarbital prior to obtaining tissue samples; ensuring that tissue samples are taken after the death of the animal, etc. Pentobarbital sodium injection is a widely accepted euthanasia method for laboratory rats due to its rapid and painless action. To perform this method, weigh the rat and calculate the dose (200 mg/kg). Prepare a sterile syringe with the calculated volume of pentobarbital sodium (100 mg/mL). Restrain the rat gently, disinfect the injection site, and administer the solution intraperitoneally in the lower right quadrant of the abdomen. Monitor the rat to ensure loss of consciousness within 1–2 min and death within 5–10 min, confirmed by the absence of vital signs.

### 2.2. GA-ONFH Modeling and Treatments

Male 6- to 8-week-old SD rats were randomly divided into the following treatment groups (10 animals per group): (i) Control; (ii) Model (lipopolysaccharide (LPS) and dexamethasone treated); (iii) continuous broad-spectrum antibiotics with Model (ABX + Model); (iv) FMT; microbiota depletion experiment [15, 16]: 1 week prior to the start of modeling, the ABX-treated rats were treated with the broad-spectrum antibiotics ampicillin (1.0 g/L), metronidazole (1.0 g/L), neomycin (0.5 g/L), and vancomycin (0.5 g/L) at doses of 160, 160, 80, and 80 mg/kg/day, respectively. These antibiotics are poorly absorbed by the intestine to allow successful targeting and depletion of intestinal commensal microbes. Water intake was measured to account for any increase in consumption due to GC treatment, and the antibiotic dose was altered to maintain the same dose among the antibiotic treatment group. Dosage and frequency of lipopolysaccharide (LPS) and GC administration: Rats received an intraperitoneal injection of LPS at a dose of 200 μg/kg once daily for 2 consecutive days. Twenty-four hours after the second LPS injection, an intraperitoneal injection of dexamethasone solution was administered at a dose of 20 mg/kg three times a week for a continuous period of 6 weeks [17]. To detect the influence of butyric acid on the femoral head, rats were given sodium butyrate (100 or 200 mg/kg/day) [18] or an equal volume of physiological salt solution once a day at the same time as dexamethasone was administered for the first time. After modeling and treatment, all rats were euthanized by overdose anesthesia with pentobarbital sodium (200 mg/kg body weight). The femoral heads were obtained and subjected to micro-CT, pathologic analysis and other further studies.

### 2.3. Fecal Microbiota Transplantation

To assess the impact of fecal microbiota transplantation (FMT) from Control group on the femoral head, feces from the Control group were collected from the rats every day during treatment [19], and gut microbiota suspensions from the Control group rats were prepared. For the preparation of gut microbiota samples, feces were resuspended in PBS (100 mg of feces per 1 mL of PBS) and centrifuged at 500 × g for 1 min to collect the supernatant (gut microbiota suspension), after which the number of bacteria was determined via a bacterial colony counting assay. The recipient microbiota-depleted dexamethasone-treated rats were fasted for 6 h before treatment, and the gut microbiota suspension was administered to the FMT group rats by oral gavage (8 × 10^9^ CFUs/kg) while using dexamethasone for modeling. Gut microbiota transplantation started at the first dexamethasone injection and was conducted every day for 6 weeks.

### 2.4. Cell Culture

Rat bone marrow mesenchymal stem cells (BMSCs) were acquired from the National Collection of Authenticated Cell Cultures (Shanghai, China) and maintained in growth medium consisting of *α*-MEM (Biological Industries, Israel) supplemented with 10% heat-inactivated fetal bovine serum (FBS; Gibco, USA) and 1% penicillin-streptomycin (Gibco, USA). Cells were incubated at 37°C in a humidified atmosphere containing 5% CO_2_ (Shanghai Jing Hong Laboratory Equipment Co., Ltd., China) [20]. Subculture was performed when cells attained 80%–90% confluency, as verified by phase-contrast microscopy (Olympus, Japan). Cells at the third passage (P3) were utilized for downstream experimental procedures. Culture medium was refreshed every 72 h.

### 2.5. 16S rDNA Sequencing

The fecal samples were collected from the –80°C freezer. A DNA extraction kit was used to extract and purify DNA from fecal samples, eliminating contaminants and impurities. After confirming the DNA concentration and quality, diluted genomic DNA was used as a template for PCR amplification of the V3~V4 region (341F: 5′-CCTAYGGGRBGCASCAG-3′, 806R: 5′-GGACTACNNGGGTATCTAAT-3′) of the 16S rDNA gene. The PCR conditions were optimized to ensure maximum yield and specificity. Purify the PCR products to remove excess primers and PCR reagents. Library construction and sequencing were performed using Illumina's library preparation kit to obtain 16S rDNA sequences of the bacterial communities in the fecal samples. Begin by conducting quality checks and filtering on the obtained 16S rDNA sequences to eliminate low-quality reads and chimeras. Subsequently, align the filtered reads with a reference 16S rDNA database to identify bacterial taxa and calculate their relative abundances.

### 2.6. Micro-CT Scan

The femoral bone samples from each group were retrieved from formalin and placed in a micro-CT scanner (Hiscan, China). Micro-CT imaging was performed using the following acquisition parameters: 80 kV, tube current of 500 μA, and isotropic voxel size of 25.0 μm. Trabecular thickness (Tb.Th) within the subchondral sclerotic region was quantified and documented using Hiscan Analyzer software to assess structural alterations in bone morphology.

### 2.7. Testing and Evaluation of Histopathology

After the intervention, the rats were euthanized, and femoral head samples were collected. The samples were fixed with 4% paraformaldehyde for 24 h. Decalcification was carried out with 10% EDTA solution for 4 weeks. The sections were made after paraffin embedding. Subsequently, H&E, Masson staining, and immunohistochemistry were performed. After staining was completed, image acquisition was performed using an upright light microscope (Eclipse E100, Nikon, Japan) and an imaging system (DS-U3, Nikon, Japan).

### 2.8. Targeted Metabolomics

10 mg/mL mixed standard stock solutions of 7 SCFAs (acetic acid, propionic acid, iso butyric acid, butyric acid, isovaleric acid, valeric acid, and caproic acid) were prepared by ether. Working solution series were prepared by appropriate dilutions of mixed standard stock solutions. Ten points calibration curve was made by adding the working solutions and an equal volume of IS solution covering a range from 0.02 to 500 μg/mL (0.02, 0.1, 0.5, 2, 10, 25, 50, 100, 250, and 500 μg/mL), and the final concentration of the internal standard was 25 μg/mL. Stock solutions were stored at −20°C before use, and working solutions were prepared when using. The samples were extracted in 50 μL of 15% phosphoric acid with 100 μL of 125 μg/mL 4-methylvaleric acid solution as the IS and 400 μL of ether. Subsequently, the samples were centrifuged at 4°C for 10 min at 12000 rpm after vortexing for 1 min, and the supernatant was transferred into the vial prior to GC‒MS analysis.

### 2.9. Cell Proliferation Assay

The Cell Counting Kit-8 (CCK-8) assay was performed to evaluate the proliferative capacity of BMSCs under different doses of sodium butyrate according to the experimental design. After 24 h, 10 μL of CCK-8 reagent was added to each well. The optical density (OD) was measured at 450 nm using a microplate reader (Bio Tek, USA), with a reference wavelength of 650 nm to subtract background interference.

### 2.10. Cell Scratch Test

The effects of different concentrations of sodium butyrate on cell migration *in vitro* were evaluated by cell scratch test. BMSCs (passage 3–5) were seeded into 6-well culture plates at a density of 5 × 10^5^ cells per well and cultured in complete medium until reaching 90%–100% confluency. The cells were incubated in serum-free medium for 24 h to eliminate the influence of serum-derived growth factors on migration. A 200 μL sterile pipette tip was used to create a straight scratch across the cell monolayer. The detached cells and debris were gently removed by washing twice with PBS. Subsequently, the original medium was replaced by complete medium containing different concentrations of sodium butyrate (0, 1, and 5 mM) in the Control groups, while the experimental group was exposed to 100 mM dexamethasone to simulate the pathological environment of GA-ONFH [21], followed by identical sodium butyrate interventions. Following a 12-h incubation period, cell migratory activity was monitored and imaged using an inverted phase-contrast microscope (Olympus, Japan). The extent of migration was quantified by analyzing captured images with ImageJ software.

### 2.11. Osteogenic Differentiation Assay

BMSCs were plated at a density of 2.5 × 10^4^ cells per well in 12-well culture plates and maintained in growth medium for 24 h. Afterward, the medium was substituted with osteogenic differentiation medium, consisting of complete medium supplemented with 50 μg/mL ascorbic acid, 10 mM *β*-glycerol sodium phosphate, and 100 nM dexamethasone. The cells were maintained under osteogenic induction conditions for 14 days, with the induction medium refreshed every 2 days. During the induction period, BMSCs in the Control groups were treated with different concentrations of sodium butyrate (0, 1, and 5 mM) in the Control groups, while the experimental group was exposed to 100 mM dexamethasone to simulate the pathological environment of GA-ONFH, followed by identical sodium butyrate interventions. To assess osteogenic differentiation potential, alkaline phosphatase (ALP) activity was evaluated on day 14 post-induction using a standardized staining protocol. Cells were gently washed with phosphate-buffered saline to remove residual medium, fixed with 4% paraformaldehyde for 20 min at room temperature, and rinsed three times with distilled water. Subsequently, cells were incubated with BCIP/NBT substrate solution for 30 min in the dark. Image-based semi-quantification: Stained cultures were photographed under an inverted fluorescence microscope (OLYMPUS CKX53, Japan). ALP-positive regions were identified as dark purple deposits under phase-contrast microscopy. The integrated optical density (IOD) of ALP-positive areas in five random fields per well was analyzed using ImageJ software with a fixed threshold to exclude background signals.

### 2.12. RNA-Seq

Total RNA was extracted from two groups of cells after 24 h of intervention by vehicle or sodium butyrate in BMSCs. Total RNA was isolated from experimental and control cell samples using TRIzol reagent (Invitrogen, Carlsbad, CA, USA), followed by RNA integrity assessment via Agilent 2100 Bioanalyzer (RIN > 7.0). Strand-specific RNA-seq libraries were prepared with the Illumina TruSeq Stranded mRNA Library Prep Kit, including poly (A) selection, RNA fragmentation, cDNA synthesis, adapter ligation, and PCR amplification. Libraries were quantified using Qubit and sequenced on an Illumina NovaSeq 6000 platform (LC-Bio Technology Co., Ltd., Hangzhou, China) with 150 bp paired-end reads. Raw sequencing data underwent quality control with FastQC, followed by adapter trimming and low-quality base removal using Trimmomatic (sliding window of 4 bases with a minimum quality threshold of 20). Functional annotation and pathway enrichment analysis were performed using clusterProfiler, employing Gene Ontology (GO) and Kyoto Encyclopedia of Genes and Genomes (KEGG) databases with hypergeometric testing (*p* < 0.05) [22].

### 2.13. Western Blot

BMSCs were harvested and homogenized in ice-cold RIPA lysis buffer via centrifugation at 12,000 × g for 15 min at 4°C. Protein concentrations were determined using a bicinchoninic acid (BCA) assay kit (Beyotime Biotechnology, China) according to the manufacturer's protocol. Equal amounts of protein lysates (20 μg per lane) were resolved by 10% sodium dodecyl sulfate-polyacrylamide gel electrophoresis (SDS-PAGE) and electrophoretically transferred onto polyvinylidene fluoride (PVDF) membranes at 80 V for 100 min. Membranes were blocked with 5% non-fat dry milk in Tris-buffered saline containing 0.1% Tween-20 (TBST) for 1 h at room temperature, followed by overnight incubation at 4°C with primary antibodies (tumor necrosis factor-alpha (TNF-*α*) (Cat#PY19810, Abmart, China), interleukin-2 (IL-2) (Cat#AF5105, Affinity, China), interleukin-4 (IL-4) (Cat#AF5142, Affinity, China) and *β*-actin (66009-1-lg; Proteintech, China)). After washing, membranes were probed with horseradish peroxidase (HRP)-conjugated secondary antibodies for 1 h. Protein bands were visualized using a ChemiDoc XRS + imaging system with enhanced chemiluminescence substrate. Band intensity was quantified using ImageJ software with the Gel Analyzer plugin.

### 2.14. Immunofluorescence Staining

The sections and neurons were blocked with 5% bovine serum albumin (containing 0.3% Triton X-100) and then incubated with the following primary antibodies at 1 : 200 overnight: TNF-*α* (Cat#PY19810, Abmart, China), IL-2 (Cat#AF5105, Affinity, China), IL-4 (Cat#AF5142, Affinity, China). Subsequently, the sections were incubated with goat anti-rabbit (Abways, China) secondary antibodies at a dilution of 1 : 300 for 1 h, followed by DAPI staining. Fluorescent signals were acquired using an inverted fluorescence microscopy system (Olympus, Japan) equipped with a CCD camera. High-resolution images were captured at 10× magnification to ensure precise visualization of cellular structures.

### 2.15. Statistical Analysis

GraphPad Prism 9.0 was used for data analysis. The results are expressed as the mean ± standard deviation (x±s). Independent sample *T* test was used for comparisons between two groups. Group comparisons were assessed with one-way ANOVA, and *p* < 0.05 was regarded as statistically significant.

## 3. Results

### 3.1. Modeling of GA-ONFH Changes the Structure of the Gut Microbiota in Rats

**Figure 1:**
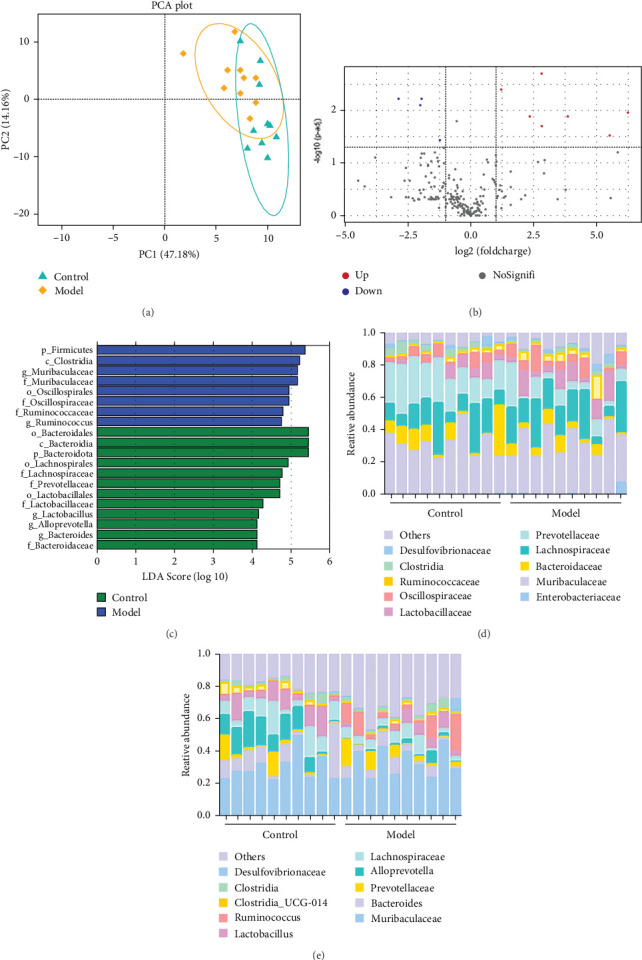
Difference in gut microbiota between Control group and GA-ONFH Model group (*n* = 10). (A) Principal component analysis. (B) Volcano plots of the gut microbiota gene profiles in Control group and Model group. (C) Histogram of the distribution of LDA effect size values. (D) Species composition abundance map of family level. (E) Species composition abundance map of genus level. GA-ONFH, glucocorticoid-associatedosteonecrosis of the femoral head.

We used 16S rDNA sequencing analysis to examine the gut microbiota composition in each group. *β*-Diversity analysis (Figure 1A), as reflected in the principal coordinates analysis (PCoA), indicated a distinct clustering pattern between the Model andControl groups, suggesting compositional differences in microbial communities. However, *α*-diversity indices did not show significant differences between groups, indicating that overall microbial richness and evenness remained unchanged.

At the family level (Figure 1C,D), in comparison to those in the Control group, the abundances of *Lachnospiraceae*, *Prevotellaceae*, *Lactobacillaceae*, and *Bacteroidaceae* were significantly downregulated in the Model group. Conversely, the abundance of *Muribaculaceae*, *Oscillospiraceae*, and *Ruminococcaceae* was significantly upregulated, and these differences were statistically significant (*p* < 0.05).

At the genus level (Figure 1C,E), in comparison to those in the Control group, the abundances of *Lactobacillus*, *Alloprevotella*, and *Bacteroides* were significantly downregulated in the Model group. Conversely, the abundance of *Muribaculaceae* and *Ruminococcus* was significantly upregulated (*p* < 0.05). This indicates that the relative abundance of the gut microbiota changed in the GA-ONFH Model group compared with the Control group.

### 3.2. Fecal Microbiota Transplantation Alleviate the Progress of GA-ONFH and Regulate the Expression of TNF-*α* and IL-2

**Figure 2:**
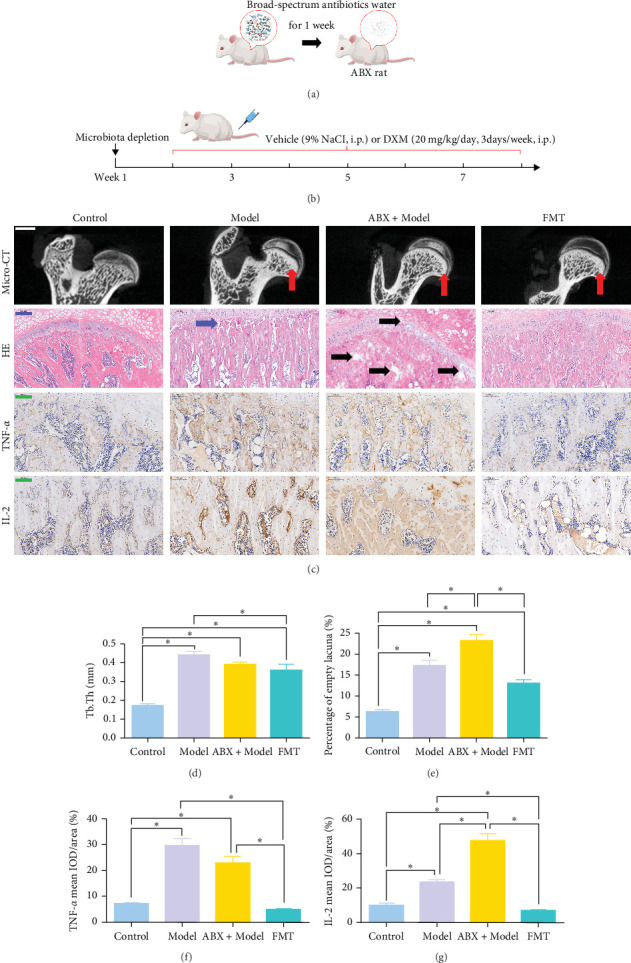
FMT alleviate the progress of GA-ONFH and decrease the expression of TNF-*α* and IL-2. (A) Microbiota depletion experiment. (B) Schematic diagram of the experimental design for the microbiota depletion experiment and GA-ONFH modeling. (C) Representative images of micro-CT (scale bars, 500 μm), HE staining (scale bars, 100 μm) and immunohistochemistry (scale bars, 100 μm). Red arrow showed subchondral sclerosis band. Blue arrow showed microfractures in subchondral sclerosis band. Black arrow showed necrotic regions in subchondral sclerosis band. (D–G) Quantification of Tb. Th (D), empty lacuna rate (E), immunohistochemical evaluation of TNF-*α* (F) and IL-2(G). *⁣*^*∗*^*p* < 0.05. GA-ONFH, glucocorticoid-associated osteonecrosis of the femoral head; IL, interleukin; TNF-α, tumornecrosisfactor-alpha.

To explore whether gut microbiota differences influence the development of GA-ONFH, we assessed the outcomes of model establishment 6 weeks post-induction using micro-CT and HE staining. Micro-CT analysis identified a subchondral sclerosis band in the femoral head of the Model group, with a marked increase in Tb.Th compared to controls (Figure 2C, D). HE staining revealed microfractures in this sclerosis band, while antibiotic treatment before model establishment resulted in significant necrotic regions. However, rats receiving FMT intervention during modeling exhibited no necrotic areas in their femoral heads (Figure 2C, E). These findings suggest that the depletion of gut microbiota by antibiotics intensified GA-ONFH progression, whereas FMT mitigated its severity. On the other hand, localized (proinflammatory) cytokine changes are observed in clinical GA-ONFH. TNF-*α* expression was found to be significantly upregulated in the articular cartilage of patients with ONFH [23]. IL-2 was also positively associated with the clinical risk of ONFH [24]. We examined whether similar changes occurred in the animal model through immunohistochemistry. The results demonstrated the levels of TNF-*α* and IL-2 in the Model group and ABX + Model group were significantly lower than those in the Control group, which were reversed by FMT intervention (Figure 2C, F, and G).

### 3.3. FMT Corrects Dexamethasone-Induced Butyric Acid Reduction

**Figure 3:**
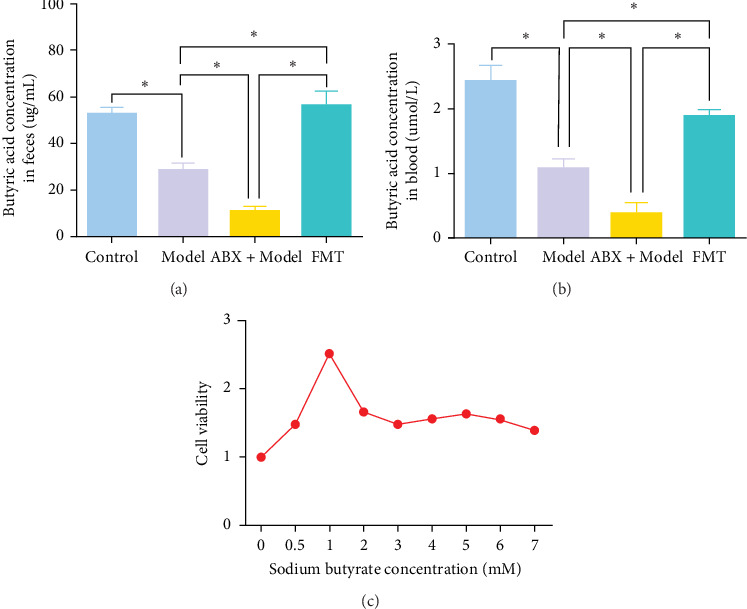
Targeted metabolomics analysis and cell proliferation assay. (A) Butyric acid concentration in feces. (B) Butyric acid concentration in blood. (C) CCK-8 analysis of BMSCs cultured with different doses of sodium butyrate. BMSCs, bone marrow mesenchymal stem cells; CCK-8, Cell Counting Kit-8. *⁣*^*∗*^*p* < 0.05.

Metabolomic analysis was conducted to identify potential bioactive metabolites that may mediate the protective effects of gut microbiota on GA-ONFH. This analysis included the evaluation of intestinal contents and serum samples from different experimental groups. The results revealed that butyrate, a short-chain fatty acid with known immunomodulatory and metabolic functions, was significantly reduced in the Model group compared to the Control group (Figure 3A,B). Furthermore, the ABX + Model group, which received broad-spectrum antibiotics before modeling, exhibited an even more pronounced decrease in butyrate levels compared to the Model group, consistent with the accelerated development of necrotic regions observed in this group. Conversely, FMT from the Control group restored butyrate levels to those comparable with the Control group, correlating with the mitigation of GA-ONFH severity in the FMT group. These findings suggest that butyrate may play a crucial role in the gut microbiota-mediated modulation of GA-ONFH.

To further investigate the potential therapeutic effects of butyrate, sodium butyrate was employed in subsequent cellular studies. A preliminary cell viability assay (CCK-8) was performed to determine the optimal concentration of sodium butyrate for treating BMSCs. Among the tested concentrations (Figure 3C), 1 mM sodium butyrate yielded the highest cell viability, indicating its potential as an effective therapeutic dose for further *in vitro* experiments.

### 3.4. Sodium Butyrate Promotes BMSCs Migration and Osteogenic Differentiation

**Figure 4:**
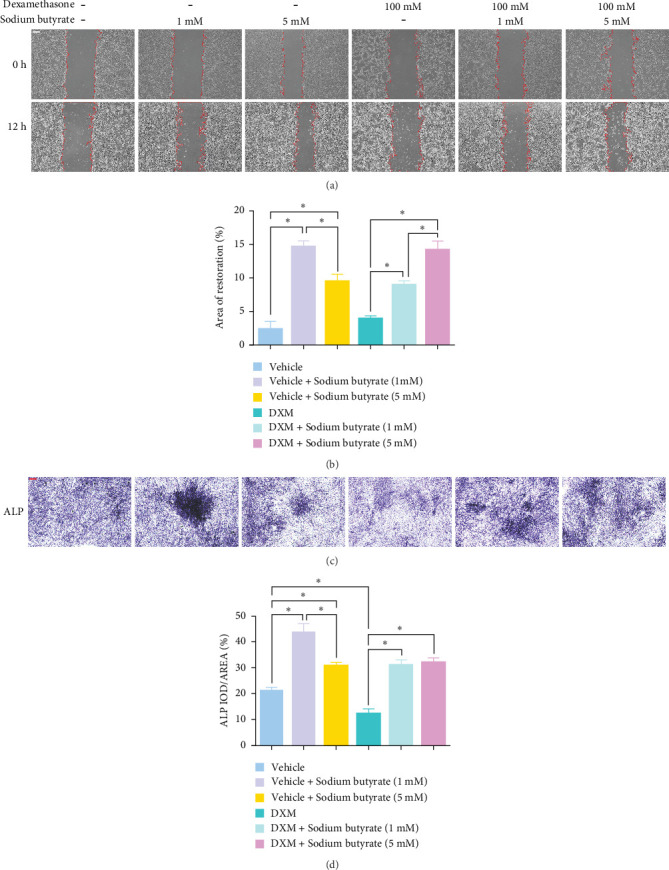
Cell scratch test and osteogenic differentiation assay. (A) Cell scratch test (scale bars, 200 μm). (B) Quantitative analysis of migration area after BMSCs cultured with different doses of sodium butyrate. (C) Osteogenic differentiation assay and ALP staining (Scale bars, 200 μm). (D) Quantitative analysis of ALP staining. ALP, alkaline phosphatase; BMSCs, bone marrow mesenchymal stem cells. *⁣*^*∗*^*p* < 0.05.

To investigate the effects of sodium butyrate on BMSCs under GA-ONFH conditions *in vitro*, we performed cell migration and osteogenic differentiation assays. BMSCs were treated with varying concentrations of sodium butyrate (0, 1, and 5 mM) in the Control group, while the experimental group was exposed to 100 mM DXM to simulate the pathological environment of GA-ONFH, followed by identical sodium butyrate interventions. The cell migration assay (Figure 4A,B) revealed that DXM alone did not significantly affect BMSCs migration. However, sodium butyrate treatment at all concentrations promoted BMSCs migration in both groups. Notably, in the Control group, the highest migration rate was observed with 1 mM sodium butyrate, while in the experimental group, 5 mM sodium butyrate elicited the most pronounced enhancement. ALP staining (Figure 4C,D), conducted to evaluate osteogenic differentiation, showed that DXM reduced BMSCs osteogenic activity. In contrast, 1 mM sodium butyrate in the Control group demonstrated the most significant promotion of osteogenic differentiation. Moreover, both 1 and 5 mM sodium butyrate effectively reversed the DXM-induced suppression of osteogenic differentiation, with no significant difference in their osteogenic effects. These findings indicate that sodium butyrate promotes both migration and osteogenic differentiation of BMSCs, potentially counteracting the detrimental effects of DXM, and underscore its potential therapeutic role in GA-ONFH.

### 3.5. Sodium Butyrate Modulates the Immune System and Cytokine Genes in BMSCs

**Figure 5:**
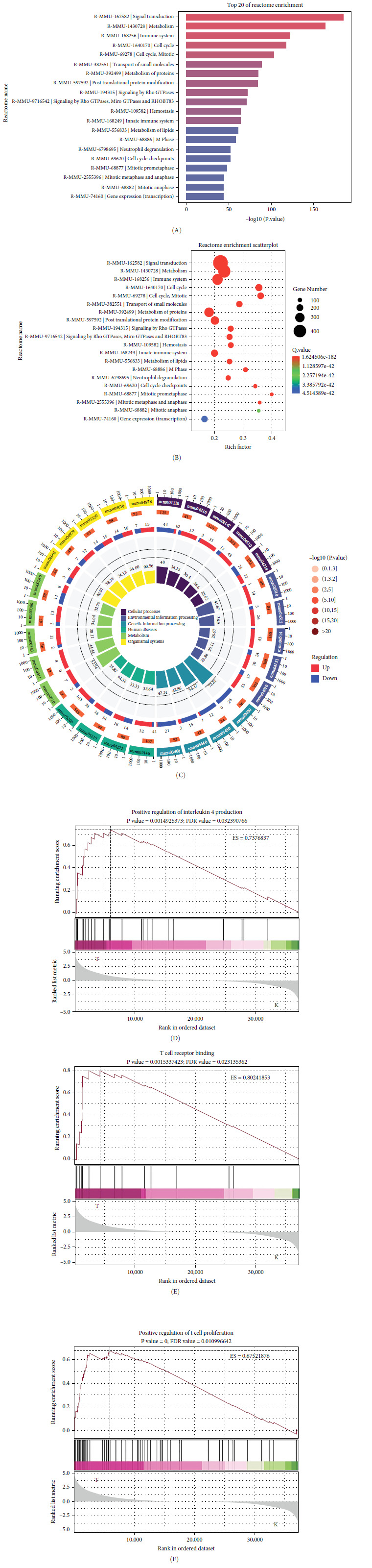
RNA-sequencing. (A) Top 20 of functional genome enrichment in sodium butyrate-treated BMSCs. (B) Functional genome enrichment scatterplot in sodium butyrate-treated BMSCs. (C) Loop circos of KEGG functional genome enrichment analysis. (D) GSEA of positive regulation of IL-4 production. (E) GSEA of T cell receptor binding. (F) GSEA of positive regulation of T cell proliferation. BMSCs, bone marrow mesenchymal stem cells; GSEA, gene set enrichment analysis; IL, interleukin; KEGG, Kyoto Encyclopedia of Genes and Genomes.

To elucidate the molecular mechanisms underlying the effects of sodium butyrate on BMSCs, we conducted RNA-sequencing (RNA-seq) analysis on sodium butyrate-treated BMSCs. Gene enrichment analysis revealed that sodium butyrate significantly modulated the expression of genes associated with the immune system and innate immune system (Figure 5A,B). Specifically, 255 immune system-related genes were upregulated, while 96 were downregulated; similarly, 149 innate immune system-related genes were upregulated, and 50 were downregulated. Furthermore, gene set enrichment analysis (GSEA) demonstrated a marked enrichment of gene sets related to the positive regulation of IL-4 production (Figure 5D), T cell receptor binding (Figure 5E), and the positive regulation of T cell proliferation (Figure 5F) in sodium butyrate-treated BMSCs, in contrast to relatively low expression levels observed in the Control group. These findings suggest that sodium butyrate exerts immunomodulatory effects *in vitro*, potentially through the upregulation of genes that promote T cell proliferation and IL-4 expression, highlighting its role in influencing immune signaling pathways in BMSCs.

### 3.6. Sodium Butyrate Regulates the Expression of TNF-*α*, IL-2, and IL-4 in BMSCS In *Vitro*

**Figure 6:**
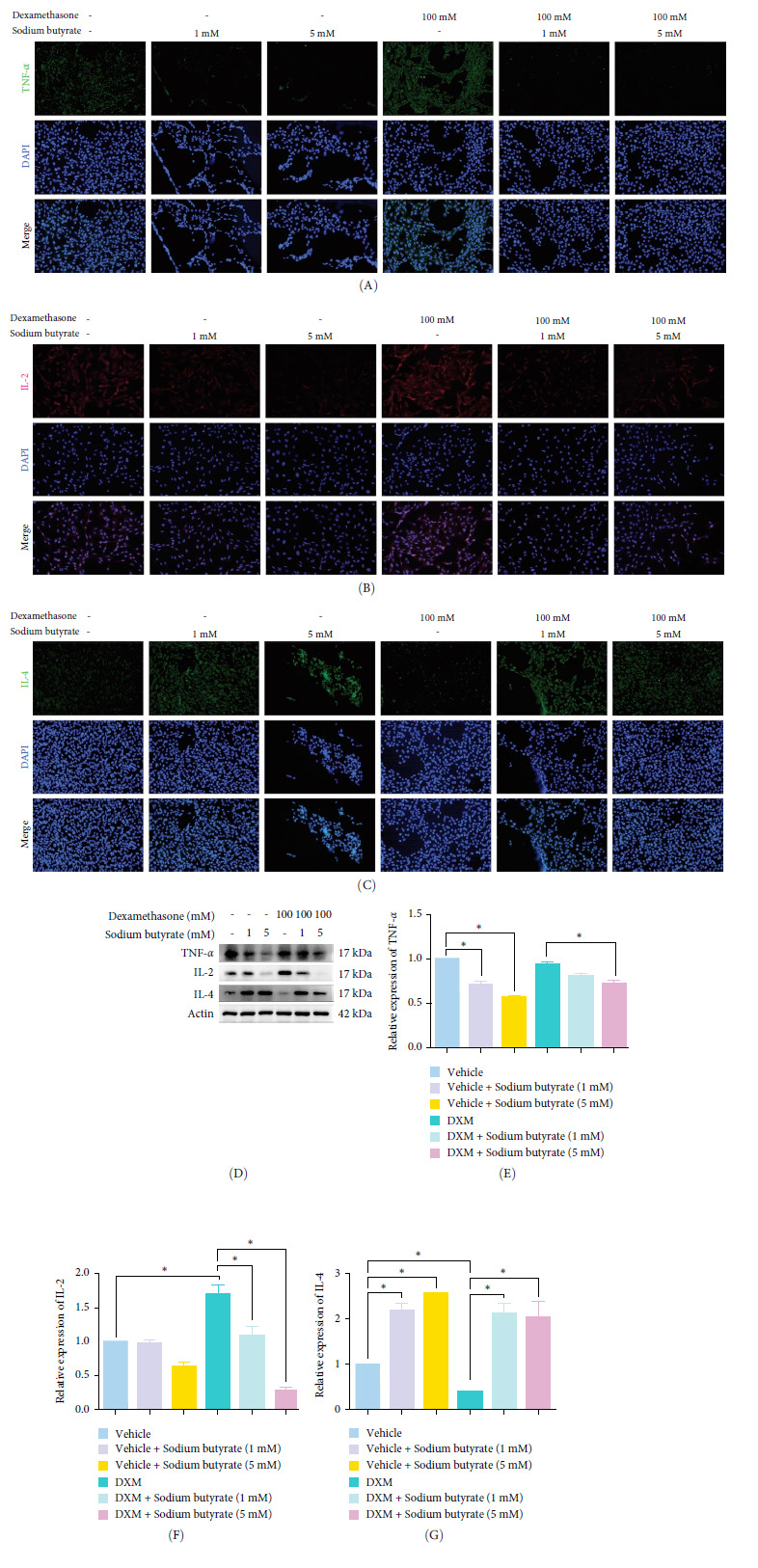
The expression of TNF-*α*, IL-2, and IL-4 in BMSCs treated with different doses of sodium butyrate. GC -: DXM 0 mM; GC +: DXM 100 mM; SCFA -: sodium butyrate 0 mM; SCFA +: sodium butyrate 1 mM; SCFA ++: sodium butyrate 5 mM. (A–C) Immunofluorescence staining of TNF-*α* (A), IL-2(B) and IL-4(C) in BMSCs. (D to G) Western blot (D) and quantitative analysis of TNF-*α* (E), IL-2(F), and IL-4(G) in BMSCs. *⁣*^*∗*^*p* < 0.05. BMSCs, bone marrow mesenchymal stem cells; GC, glucocorticoids; IL, interleukin; SCFA, short-chain fatty acids; TNF-*α*, tumor necrosis factor-alpha.

To investigate the effects of sodium butyrate on cytokine expression in BMSCs under GA-ONFH conditions *in vitro*, we conducted a comparative study employing different concentrations of sodium butyrate (0, 1, and 5 mM). In the Control groups, BMSCs were treated solely with sodium butyrate, while the experimental groups were exposed to 100 mM DXM to simulate the pathological environment of GA-ONFH, followed by identical sodium butyrate interventions. Western blot analysis revealed that DXM had no significant impact on the expression of TNF-*α*; however, it markedly increased IL-2 expression and decreased IL-4 expression. Treatment with 5 mM sodium butyrate notably downregulated TNF-*α* expression in both the control and experimental groups. In contrast, neither 1 nor 5 mM sodium butyrate significantly altered IL-2 expression under baseline conditions, yet both concentrations effectively reversed the DXM-induced upregulation of IL-2. Additionally, sodium butyrate at 1 and 5 mM concentrations promoted IL-4 expression in BMSCs and counteracted the downregulation of IL-4 caused by DXM exposure. These findings suggest that sodium butyrate exerts modulatory effects on cytokine profiles in BMSCs, potentially mitigating DXM-induced dysregulation of IL-2 and IL-4 expression while suppressing TNF-*α*, indicating its therapeutic potential in the context of GA-ONFH (Figure 6).

### 3.7. Sodium Butyrate Alleviates the Progress of GA-ONFH and Regulates the Expression of Cytokines in *Vivo*

**Figure 7:**
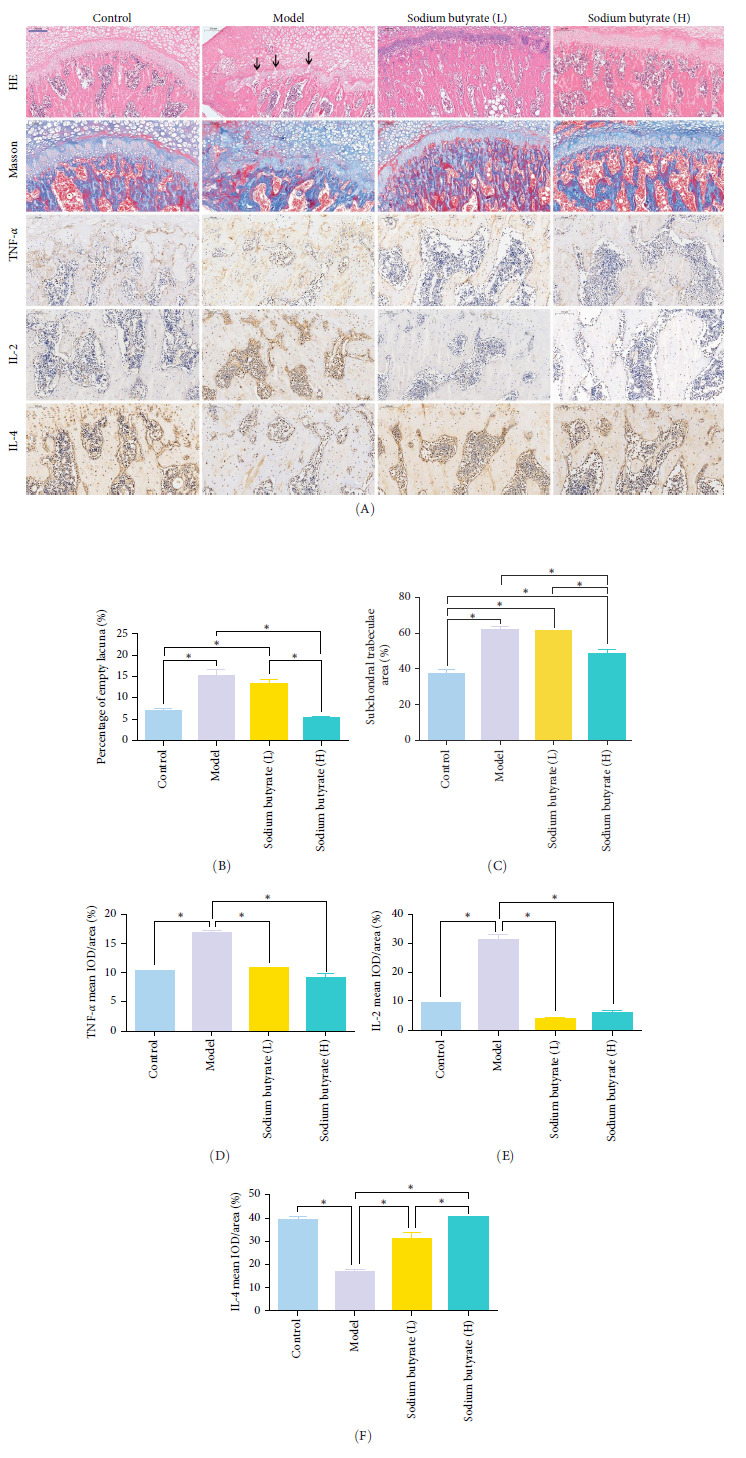
Sodium butyrate alleviates the progress of GA-ONFH and regulates the expression of cytokines *in vivo*. (A) Representative images of HE (scale bars, 200 μm), masson staining (scale bars, 200 μm) and immunohistochemistry (scale bars, 100 μm). Black arrow showed discontinuity of the epiphyseal plate in Model group. (B to F) Quantification of empty lacuna rate (B), Subchondral trabeculae area (C), immunohistochemical evaluation of TNF-*α* (D), IL-2 (E), and IL-4 (F). *⁣*^*∗*^*p* < 0.05.GA-ONFH, glucocorticoid-associated osteonecrosis of the femoral head; IL, interleukin; TNF-α, tumor necrosis factor-alpha.

The *in vivo* effects of sodium butyrate on the progression of GA-ONFH were evaluated using histopathological and immunohistochemical analyses. Rats were treated with either a low dose (100 mg/kg) or high dose (200 mg/kg) of sodium butyrate during the 6-week modeling period, alongside Model and Control groups. Histopathological examination revealed that the Model group exhibited a significant increase in empty lacunae ratio and trabecular area compared to the Control group, alongside characteristic disruptions such as discontinuity of the epiphyseal plate observed through HE staining. Conversely, while the low-dose sodium butyrate group exhibited no significant difference in empty lacunae rates or trabecular bone area compared to the Model group, both sodium butyrate-treated groups displayed preserved epiphyseal continuity and an absence of microfracture features, highlighting the protective effects of sodium butyrate against structural damage (Figure 7A–C).

Immunohistochemical analyses (Figure 7A, D–F) revealed altered cytokine express in the femoral head across groups. The expression of TNF-*α* and IL-2 was markedly elevated in the Model group compared to the Control group, reflecting a pro-inflammatory state associated with GA-ONFH. Both low- and high-dose sodium butyrate groups showed reduced expression of TNF-*α* and IL-2 relative to the Model group, with no significant difference between the two sodium butyrate-treated groups. Conversely, IL-4 expression, which was significantly decreased in the Model group compared to the Control group, was restored in both sodium butyrate-treated groups. Importantly, high-dose sodium butyrate intervention achieved IL-4 levels comparable to the Control group and significantly higher than those in the low-dose sodium butyrate group. These findings suggest that sodium butyrate mitigates the progression of GA-ONFH by preserving trabecular integrity and modulating cytokine expression, particularly through suppression of proinflammatory mediators and enhancement of anti-inflammatory IL-4 expression.

## 4. Discussion

GA-ONFH remains a significant clinical challenge due to its multifactorial pathogenesis and limited therapeutic options. Its pathogenesis is still not a recognized and clear conclusion. Current research highlights the critical role of impaired vascularization [25], osteocyte apoptosis [26], and imbalanced bone remodeling [27, 28] in the progression of GA-ONFH. Despite advancements in understanding the molecular mechanisms, the long-term use of GCs—a widely prescribed class of drugs for their potent anti-inflammatory and immunosuppressive properties—continues to pose a substantial risk for developing GA-ONFH. These agents disrupt bone homeostasis by impairing osteogenic differentiation, promoting adipogenesis, and triggering inflammatory cascades, underscoring the urgent need for targeted interventions that mitigate these adverse effects while preserving therapeutic benefits.

Notably, previous experimental studies have largely overlooked the local cytokine milieu in GA-ONFH, which plays a pivotal role in regulating immune responses and bone metabolism. Cytokines, as key mediators of cellular communication, are intricately involved in maintaining bone homeostasis by orchestrating the balance between osteoblast-mediated bone formation and osteoclast-driven bone resorption. Dysregulated cytokine signaling has been implicated in the pathogenesis of several bone-related diseases, including osteoporosis, osteoarthritis, and osteonecrosis [29, 30]. Proinflammatory cytokines, such as TNF-*α* and IL-2, have been demonstrated to promote cell proliferation and angiogenesis while inhibiting osteogenesis. Specifically, TNF-*α* increases osteoblast apoptosis and enhances the expression of receptor activator of nuclear factor kappa-B ligand (RANKL), indirectly leading to elevated osteoclast differentiation and activity while suppressing osteoclast apoptosis [31]. In addition, TNF-*α* was observed to be significantly upregulated in the articular cartilage of ONFH patients, which may be relevant to the pathogenesis of ONFH [23]. A study assessing the association between 41 proinflammatory cytokines and osteonecrosis by Mendelian randomization identified IL-2 as causally associated with osteonecrosis risk [24]. Conversely, anti-inflammatory cytokines, such as IL-4, are known to reduce the infiltration of M1 macrophages and sustain the activation of M2 macrophages, thereby limiting inflammation and minimizing osteocyte apoptosis [32]. Furthermore, IL-4 may participate in the recruitment of osteoblasts within bone tissue, highlighting its critical role in cytokine-mediated regulation of bone resorption and healing [33].

Our earlier clinical research highlighted a link between gut microbiota alterations and the development of GA-ONFH. Specifically, patients with GA-ONFH showed a marked decrease in *Lachnospiraceae* abundance, along with elevated serum levels of inflammation-related factors such as IL-17A, IL-33, and TNF-*α* [17]. Based on our previous clinical studies, this study aimed to address this gap by investigating the effects of gut microbiota or its metabolic products on GA-ONFH through a cytokine-centric lens, utilizing multi-omics approaches and complementary *in vitro* and *in vivo* models. In the animal model of GA-ONFH, a marked dysbiosis was observed, characterized by a significant reduction in the abundance of potentially beneficial bacterial families such as *Lachnospiraceae*, *Prevotellaceae*, *Lactobacillaceae*, and *Bacteroidaceae*, accompanied by an increased presence of conditionally pathogenic bacteria, including *Muribaculaceae*. In addition, disrupting the native gut microbiota of rats with antibiotics prior to model establishment significantly accelerated GA-ONFH progression. In contrast, administering FMT during modeling slowed its development. These findings point to a potential connection between gut microbial imbalance and GA-ONFH worsening. The gut microbiota has been increasingly recognized as a critical regulator of various physiological processes and is associated with the pathogenesis of numerous diseases, including metabolic [34, 35], autoimmune [36], and inflammatory disorders [37]. It exerts significant influence on host immunity by modulating inflammatory responses through its metabolites and interaction with immune cells. In this study, we observed significant changes in the gut microbiota of GA-ONFH model rats. Since the microbiota itself cannot breach the intestinal barrier, we speculate that gut microbial metabolites are likely mediators of GA-ONFH progression.

Metabolomic profiling further identified butyrate as a key microbial-derived metabolite with therapeutic potential, as its levels were significantly reduced in the GA-ONFH Model group and ABX + Model group, but restored in FMT group. Our experiments *in vitro* demonstrate that DXM treatment induces significant alterations in cytokine expression, notably upregulating IL-2 and downregulating IL-4, alongside impairing osteogenic differentiation and migration of BMSCs, whereas sodium butyrate reverses DXM-induced dysregulation of IL-2 and IL-4 while downregulating TNF-*α* expression. Notably, DXM has been used as an anti-inflammatory agent in previous studies to reduce cytokine expression during inflammatory responses. TNF-*α* expression is mainly regulated by NF-*κ*B signaling, and GCs usually inhibit NF-*κ*B signaling in immune cells thereby reducing TNF-*α* expression [38]. However, in our study, DXM treatment did not significantly alter TNF-*α* expression in BMSCs, suggesting that its regulatory effects on proinflammatory cytokines may be cell-type dependent. In contrast, DXM significantly upregulated IL-2 expression. This observed IL-2 upregulation may be attributed to two mechanisms. First, DXM may exert bidirectional immunomodulatory effects, enhancing IL-2 production in BMSCs within the local microenvironment, thereby triggering inflammatory responses. Second, DXM-mediated suppression of osteoblast differentiation may disrupt bone remodeling homeostasis, resulting in the release of damage-associated molecular patterns (DAMPs) [39]. These DAMPs may further activate innate immune pathways within joint tissues [40]. Furthermore, DXM markedly reduced IL-4 expression, consistent with previous reports of glucocorticoid-mediated IL-4 suppression. These findings highlight DXM's differential regulatory effects on cytokine expression in BMSCs and suggest that while immunosuppressive overall, its cytokine-specific effects are context-dependent, potentially contributing to the altered immune microenvironment in GA-ONFH.

Further *in vivo* experiments demonstrated that sodium butyrate alleviates GA-ONFH progression in rats and reverses glucocorticoid-induced dysregulation of TNF-*α*, IL-2, and IL-4. Postbiotics SCFAs have been shown to play a crucial role in immune regulation [41]. Among them, butyrate has been demonstrated to reduce the levels of the inflammatory cytokine IL-6 in a dose-dependent manner, significantly decrease osteoclast formation and resorption activity, and promote bone healing by showing a tendency for increased calcium deposition in MSC cultures [42]. In this study, we confirmed that sodium butyrate effectively mitigated the progression of GA-ONFH by reversing glucocorticoid-induced dysregulation of TNF-*α*, IL-2, and IL-4 both *in vivo and in vitro*, promoting T cell proliferation, improved the local inflammatory environment, and enhanced the osteogenic differentiation and migratory capacity of BMSCs.

These findings underscore the therapeutic potential of targeting the gut microbiota or its metabolites in mitigating the cytokine-driven pathophysiological alterations associated with GA-ONFH. By modulating immune responses and restoring bone homeostasis, sodium butyrate exemplifies a promising avenue for future therapeutic strategies. Further research should explore the precise molecular pathways underlying these effects, particularly the interplay between gut microbiota-derived metabolites, local cytokine networks, and bone tissue remodeling in GA-ONFH. This integrative approach holds promise for the development of innovative treatments aimed at reducing glucocorticoid-related adverse effects and improving patient outcomes.

## 5. Conclusion

This study highlights the critical role of gut microbiota and its metabolites in the progression and mitigation of GA-ONFH. Dysbiosis characterized by reduced beneficial bacteria and decreased butyrate levels was linked to increased bone damage and inflammation. Restoration of butyrate through FMT or sodium butyrate supplementation alleviated osteonecrosis by modulating inflammatory cytokines, reducing TNF-*α* and IL-2, and increasing IL-4 expression. Histopathological improvements further confirmed the therapeutic potential of butyrate. These findings underscore butyrate's promise as a novel intervention for GA-ONFH, warranting further investigation for clinical application.

## Data Availability

The data that support the findings of this study are available from the corresponding author upon reasonable request.

## Nomenclature

GA-ONFH: Glucocorticoid-associated osteonecrosis of the femoral head
BMSCs: Bone marrow mesenchymal stem cells
Tb.Th: Trabecular thickness
SD: Sprague–Dawley
LPS: Lipopolysaccharide.
